# Characterization of Glass-Ceramic Sealant for Solid Oxide Fuel Cells at Operating Conditions by Electrochemical Impedance Spectroscopy

**DOI:** 10.3390/ma13214702

**Published:** 2020-10-22

**Authors:** Roberto Spotorno, Marlena Ostrowska, Simona Delsante, Ulf Dahlmann, Paolo Piccardo

**Affiliations:** 1 Department of Chemistry and Industrial Chemistry, University of Genoa, via Dodecaneso 31, 16146 Genoa, Italy; ostrowska.marlena@gmail.com (M.O.); simona.delsante@unige.it (S.D.); paolo.piccardo@unige.it (P.P.); 2 R & D Product Division Glass, SCHOTT AG, Christoph-Dorner-Straße 29, 84028 Landshut, Germany; ulf.dahlmann@schott.com

**Keywords:** solid oxide fuel cells, sealing, EIS, glass-ceramic, crystallization, interaction

## Abstract

A commercially available glass-ceramic composition is applied on a ferritic stainless steel (FSS) substrate reproducing a type of interface present in solid oxide fuel cells (SOFCs) stacks. Electrochemical impedance spectroscopy (EIS) is used to study the electrical response of the assembly in the temperature range of 380–780 °C and during aging for 250 h at 780 °C. Post-experiment analyses, performed by means of X-ray diffraction (XRD), and along cross-sections by scanning electron microscopy (SEM) and energy dispersive X-ray (EDX) analysis, highlight the microstructural changes promoted by aging conditions over time. In particular, progressive crystallization of the glass-ceramic, high temperature corrosion of the substrate and diffusion of Fe and Cr ions from the FSS substrate into the sealant influence the electrical response of the system under investigation. The electrical measurements show an increase in conductivity to 5 × 10^−6^ S∙cm^−1^, more than one order of magnitude below the maximum recommended value.

## 1. Introduction

Solid oxide fuel cells (SOFCs) constitute one of the most promising technologies for alternative energy production. Such devices are able to convert the chemical energy stored in a fuel directly to electricity without intermediate steps. Such a reaction is characterized by high efficiency, which can be further increased by partially recovering the exhaust heat. Due to the high operating temperatures (600–800 °C), they do not require the use of expensive noble catalysts and various fuels can be internally reformed. Other advantages of SOFCs are the low emission of polluting species, modularity and static functioning, without noise and vibrations [1].

A single cell consists of three essential components: two porous electrodes (i.e., cathode and anode) separated by a thin and dense electrolyte. The state-of-the-art material for the anode is a nickel-YSZ cermet, while the cathode is made of perovskites such as strontium-doped lanthanum manganite (LSM) or (La, Sr) (Co, Fe)O_3_ (LSCF). The electrolyte is commonly constituted by yttria-stabilized zirconia (YSZ) [2].

One cell can produce around 1V open circuit potential and a limited amount of current, depending on its size [3]. For practical applications, cells are connected in series forming a stack, which requires some additional components such as interconnects, frames and sealants, depending on their design [4]. The most common stack geometry is planar, where the cells are placed one on top of the other, connected through interconnects. These additional elements are commonly designed with a wavy section forming channels for the flow of reactant gases. The parts in contact with the electrodes provide the electrical connection between the cathode of one cell and the anode of the following one [5]. Ferritic stainless steel (FSS) is the most used material for interconnects being economical and characterized by a low coefficient of thermal expansion (CTE), compatible with other components of the stack. Additionally, it forms a protective chromium oxide layer on the surface, which prevents it from high-temperature corrosion [6,7]. The same material can be used for building the frames, which have the aim of keeping separated the anodic compartment from the cathodic one, avoiding mixing of the reactants.

**Figure 1:**
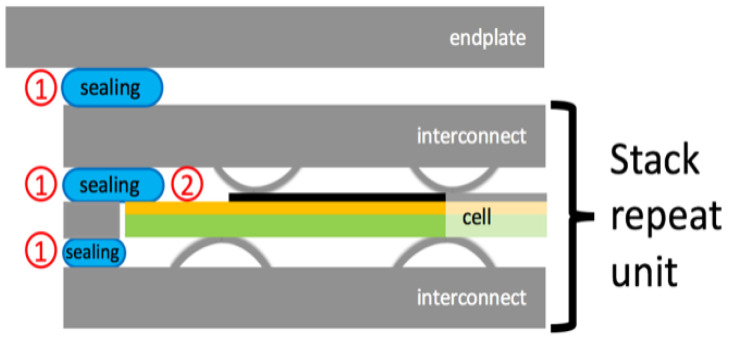
Detail of a stack scheme showing the position of sealing and its interfaces. ① indicates the sealing portions in contact with steel at both sides; ② indicates the sealing portions in contact with steel on one side and ceramic on the other side.

Sealants are used to join the abovementioned components, keeping them electrically separated to avoid short circuits (Figure 1). They also provide gas tightness, preventing leakages and mixing of the reactants. Sealant materials have to be, therefore, electrically insulating and gastight. Additionally, they have to be chemically and mechanically compatible with the adjoining elements to ensure stable bonds and avoid interactions leading to local changes of the sealant properties, causing cracks or delamination [8,9]. 

Glass and glass-ceramic materials are often indicated to be optimal solutions for sealing SOFCs stacks because of their possibility to achieve good adhesion to the metal substrates, gas tightness, high electrical resistance, ease of fabrication and application and competitive costs [10,11,12,13].

The selection of the glass or glass-ceramic composition is driven by its CTE and transition temperature (Tg). The first one should be compatible with the adjoining components (i.e., interconnect and electrolyte) in order to avoid the introduction of mechanical stresses and reduce risks of failures caused by thermal shocks [8,12,13,14,15]. The Tg must be close to the stack working temperature since the glass has to soften, providing a proper seal but maintaining a sufficient rigidity [16]. 

The materials involved in cells and stacks assembly are subjected to several degradation mechanisms due to the exposure to the cell reactants and high temperatures characterizing the stack operating conditions [17]. Some of them are caused by phase changes, migration of elements, reactions between the components, formation of cracks or short-circuiting [18,19]. Undesirable phases can be formed at the sealant/substrate interface as a result of diffusion processes and chemical reactions which can lead to the local deterioration of the glass properties [20,21,22,23].

Furthermore, some glass-ceramics undergo progressive crystallization at SOFC operating temperature, leading to alterations of the residual glass composition. The electrical properties of glass-ceramic depend on the amount and distribution of such phases in the bulk or at the interface with the steel [24,25]. 

Sealants are commonly characterized by post-experiment techniques on operated stacks to evaluate their microstructure and interaction with the neighboring materials. In several studies, their electrical properties are measured during aging, using sample designs allowing DC resistance measurements at SOFC working conditions. The application of electrochemical impedance spectroscopy (EIS) for the characterization of glass-ceramic materials could constitute a powerful tool to study the effects of bulk or interfacial changes in the sealant [26,27,28]. The implementation of such a technique can also be used to monitor, in a non-destructive way, the state of the sealant in the stacks. 

In this work, a commercially available glass-ceramic composition was applied on FSS substrates assembling metal/glass/metal samples, thus reproducing the situation of portions 1 in Figure 1. Such samples are suitable to characterize the evolution of bulk and the interactions of the involved materials at the interfaces.

EIS was applied to characterize the electrical response of the assembly in the range of 380–780 °C and its evolution during aging for 250 h at 780 °C, considering the average temperature in an SOFC stack. Such set of measurements allowed to identify different impedance contributions and to evaluate the influence of glass crystallization and interaction with the substrate on its electrical properties. An equivalent circuit describing the system has been proposed and used to fit the measured data. Post-experimental analyses were performed on the cross-sections of samples by means of scanning electron microscopy (SEM) and energy dispersive X-ray (EDX) analysis, identifying the major microstructural changes responsible for the electrical properties’ evolution. 

## 2. Materials and Methods

### 2.1. Preparation of Samples

A commercially available barium silicate glass-ceramic composition (G018-311, SCHOTT AG, Mainz, Germany) was applied on FSS plates constituting metal/glass/metal and metal/glass samples. The first type was used for the electrical characterization and post-experiment investigation on the cross-sections. Metal/glass samples were used for the post-experiment X-ray diffraction.

The steel substrates preparation consisted of cutting 6 × 6 × 0.5 mm^3^ plates from sheets of AISI 441 (composition in Table 1). The plates were then ultrasonically cleaned (600 W, 40 kHz) for 5 min in acetone and 5 min in deionized water to avoid contaminations at the interfaces. No surface changes were applied after the cold rolling carried out by the steel producer.

Glass-ceramic material, provided as powder, was mixed with an acrylic-based binder provided by the sealant producer. The powder/binder ratio was approximately 10:1 in weight, adjusted to obtain a paste in order to ensure proper handling and application to the substrate. Such mixture was homogenized using a mechanical blender and kept in an ultrasonic bath (600 W, 40 kHz) for 5 min to remove gas bubbles formed during the mixing step. The paste was then applied on a substrate using a spatula to obtain a homogeneous layer. A second steel plate was then applied over the glass-ceramic paste obtaining the metal/glass/metal samples.

Each assembly was then thermally treated for 1 h at 850 °C in static air to remove the binder, sinter the glass-ceramic powder and join it to the substrates. The heating rate was 5 °C∙min^−1^, while the cooling ramp was 2 °C∙min^−1^. 

Three samples of each type were prepared following this procedure and aged for different times (i.e., 100 and 250 h) before post-experiment characterization in order to investigate the effect of aging at the SOFC working conditions.

### 2.2. Electrochemical Characterization

Samples were contacted using gold meshes on steel plates and mounted in a custom testing apparatus allowing to control the temperature and perform electrical measurements using four-terminal sensing. A frequency response analyzer (SI 1260 Electrochemical Interface, Solartron Analytical, Farnborough, UK) was used to carry out the EIS measurements applying 1 V AC signal in the frequency range of 0.1–200 kHz. EIS was measured in the temperature range of 380–780 °C and during aging for 250 h at 780 °C. The complex non-linear regression least squares (CNRLS) method [29,30] was applied to fit the EIS spectra, by means of the ZView® software (Scribner Associates Inc., Southern Pines, NC, USA).

### 2.3. Post-Experiment Characterization

X-ray diffraction on aged metal/glass samples was performed by means of a diffractometer, Philips X’Pert MPD machine, in order to characterize the crystalline phases; the instrument works with a Cu anode excited to 40 kV, 30 mA and a solid state detector. A step scan mode (10° ≤ 2θ ≤ 100° range, step size of 0.03° and time per step of 4 s) was employed to record the XRD patterns. The identification of the phases was performed by using the program PowderCell [31].

Metal/glass/metal samples were cut, in order to expose their cross-sections, and prepared following the ASTM E3-11 metallographic procedure (Standard Guide for Preparation of Metallographic Specimens). Samples were coated with 5 nm of gold by magnetron sputtering and characterized using a scanning electron microscope (Zeiss EVO 40, Carl Zeiss, Oberkochen, Germany) equipped with an energy dispersive X-ray spectroscope (EDX Pentafet, Oxford Instruments, Oxfordshire, UK).

The glass-ceramic crystallinity and steel roughness ratio were estimated from back-scattered electrons (BSE) images at 5000× using the software ImageJ, version 1.49b [32]. The crystallinity was measured on four square portions of 60 × 60 µm^2^ of the glass-ceramic bulk for each sample. The roughness ratio, defined as the ratio between the actual and projected surface, was measured at the steel/sealant interface. EDX line scans at the interfaces were used to calculate the diffusion coefficient of elements from the substrates into the glass.

## 3. Results and Discussion

**Figure 2:**
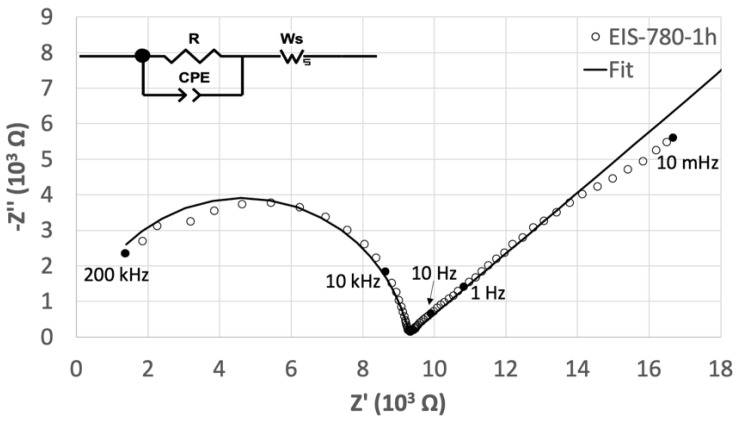
Nyquist plot of EIS measured at 780 °C (circles) and fit (solid line). The inset shows a schematic model of the equivalent electric circuit used for fitting.

The Nyquist plot (Z’’ vs. Z’) of EIS measured after one hour at 780 °C is shown in Figure 2. Two processes are visible, distinguishable by a high-frequency semicircle and a tail at low frequencies. To fit such data, an equivalent circuit (inset in Figure 2) was proposed, according to the features characterizing the Nyquist plot. The high-frequency process can be attributed to the bulk resistance and the capacitance of the sample [24,26,33]. It is usually fitted using a resistor and a capacitor connected in parallel [34], however, the semicircle shown in Figure 2 has a depressed shape with the center below the real axis. Such behavior may be the result of the heterogeneity of the system and presence of a distribution in relaxation times within the bulk response [35]. It can be fitted using a constant phase element (CPE), consisting of an empirical impedance function defined by

(1) $$Z_{CPE}=\frac{1}{Q{\left(j\omega \right)}^{n}}$$

where *j* is the imaginary unit, ω is the frequency and n and Q are frequency-independent coefficients. The time constant of the CPE is independent from the frequency when n=1 and, in that case, the element can be treated as a pure capacitor, where Q has units of a capacitance. When n<1, the time constant of the CPE becomes frequency-dependent and the element is represented as a depressed semicircle in the Nyquist plot. 

In the low-frequency range, data showed a continuous increase in the impedance values when decreasing the frequency. Such behavior can be related to the thickness of the glass. When the material, where diffusion processes occur, is thick, the lowest applied frequencies do not completely penetrate the diffusion layer, which could be considered as semi-infinite [36]. A Warburg impedance element can be used to fit the low-frequency range. This element, characterized by a frequency-dependent time constant, is commonly used to describe charge carriers diffusing through a material. It represents the solution of the one-dimensional diffusion equation of a particle and its impedance is defined by

(2) $$Z_{W}=R\cdot \frac{tanh\left[{\left(j\cdot T\cdot \omega \right)}^{P}\right]}{{\left(j\cdot T\cdot \omega \right)}^{P}}$$

with $T=L^{2}/D$, where L and D stand for the effective diffusion thickness and the effective diffusion coefficient of the particle, respectively; tanh is the hyperbolic tangent and P is a frequency-independent coefficient, related to the time constant distribution [37,38].

The deviation from the Warburg behavior, noticeable at the lowest frequencies, can be ascribed to the blocking effect of the electrodes. Such phenomenon occurs at the glass/electrode interface where the mobile ions are stopped due to their inability to diffuse through the metal. This leads to an accumulation of ions resulting in a large polarization at the interface [33,39,40].

**Figure 3:**
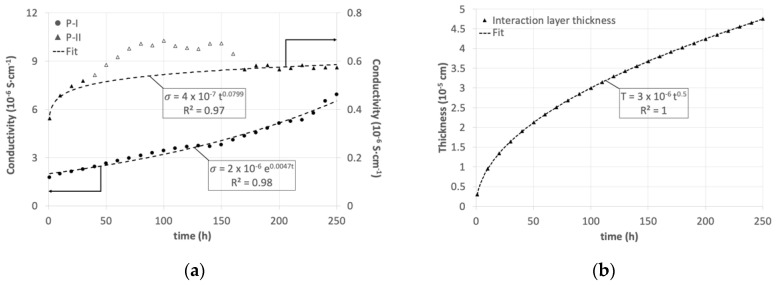

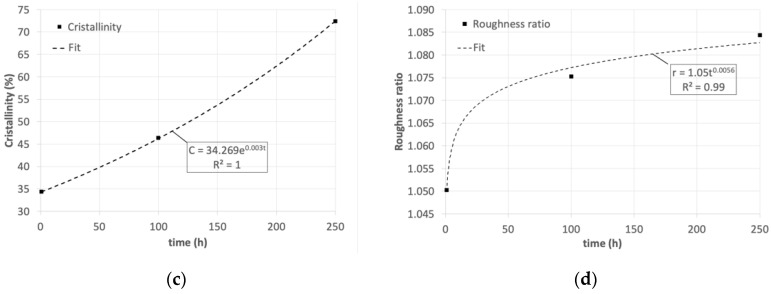
Evolution over time of (**a**) the EIS contributions; (**b**) thickness of the interaction layer; (**c**) crystallinity and (**d**) the roughness ratio. Data plotted as empty triangles in (**a**) were not taken into account for fitting the P-II trend.

When the sample was kept at 780 °C, its EIS contributions changed over time, as displayed in Figure 3a. The conductivities related to the impedance processes were calculated from their resistances using the relation

(3) $$\sigma =\frac{1}{R}\frac{l}{S}$$

where l is the thickness of the glass and S is its cross-sectional area, both measured by post-experiment characterization. 

The conductivity of the high-frequency process showed a continuous increase during the whole experiment following an exponential trend. The contribution related to the Warburg impedance followed a power law rapidly increasing in the first 50 h at 780 °C, then stabilizing for the rest of the experiment.

**Figure 4:**
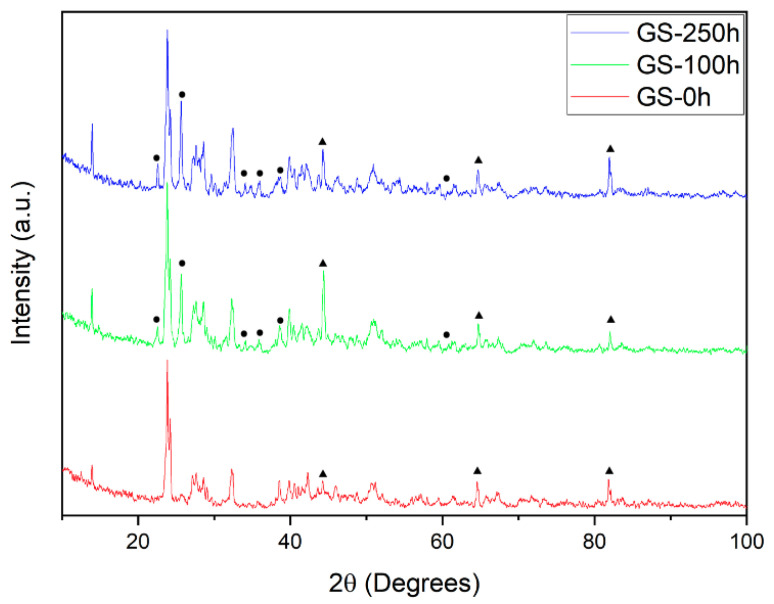
Diffraction profiles of samples after sintering (red), aged for 100 h (green) and for 250 h (blue). Peaks not related to the Ba_3_Si_2_O_8_ phase are indicated in the spectra with (▴) for the Fe-Cr alloy and (⦁) for MgCrO_4_.

**Figure 5:**
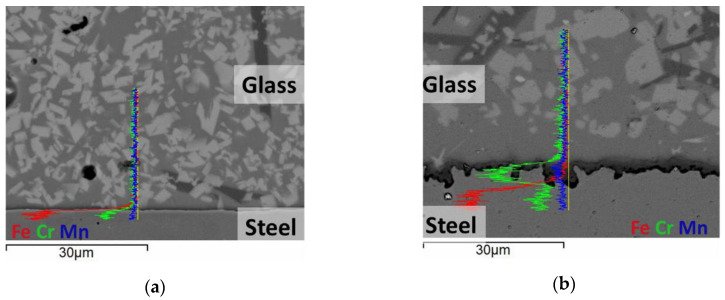
SEM-BSE cross-section image and EDX line scans of the sample (**a**) before aging and (**b**) aged for 250 h at 780 °C.

Such observations were confirmed by post-experiment characterization. The diffraction profile of the as sintered sample shows the presence of Ba_3_Si_2_O_8_ crystals and peaks related to the stainless steel substrate (Figure 4). Such crystals are visible in SEM images (Figure 5), resulting as homogeneously distributed in the glass matrix of the sealant. Comparing samples before and after aging, it was possible to observe an increase in the size of crystals. The crystallinity value continuously increased over time following an exponential trend (Figure 3c). This behavior was in agreement with the conductivity trend of the high-frequency process (P-I, Figure 3a). Such effect can be related to the enhancement of the ionic conductivity of the glass, since the formation and growth of insulant Ba_3_Si_2_O_8_ crystals increases the concentration of charge carriers in the residual glass matrix [24,25,41]. The electric conductivity of most glasses is due to mobile ions [39]. Small variations of their concentration in the residual glass lead to strong conductivity changes [40,42,43]. 

After aging, XRD showed additional peaks ascribable to the compound MgCrO_4_ which could result from the interaction of magnesium, contained in the sealant, with chromium diffused from the substrate. Such a phase was detected at the substrate/sealant interface, appearing as a dark, chromium-rich layer located between the steel and the glass (Figure 5). The formation of MgCrO_4_ at the steel/glass interface has been already reported in the literature [16,44] and indicated as a possible cause of local CTE variations. This phase is detectable by post-experiment characterization since it is stable only below 600 °C [45], thus formed by the reaction of chromium with the magnesium oxide and oxygen contained in the glass when cooling the system. Above 600 °C, MgCrO_4_ decomposes, forming MgO and ${\mathrm{Cr}}_{2}O_{3}$. Such oxides are not expected to affect the mechanical properties at the interface, MgO being already present in the glass composition, and ${\mathrm{Cr}}_{2}O_{3}$ already formed on the steel surface, as an effect of the high-temperature oxidation. However, the consequences of this reaction, due to thermal cycling and the increase in such compounds at the interface for a longer aging time, need to be verified.

On the other hand, the presence of chromium oxide could locally enhance the electrical conductivity at the operating temperature having a semiconducting behavior [46].

The EDX line scan (Figure 5) highlighted also iron diffusion within the glass, however no related phases were detected by XRD. 

The diffusion coefficient of ions from the steel into the glass-ceramic was calculated using the EDX profile of the elements. The solution to Fick’s law in one dimension for the diffusion in a semi-infinite medium was used [47]:

(4) $$\left(\frac{C\left(x\right)-C_{0}}{C_{i}-C_{0}}\right)=\frac{1}{2}erfc\left(\frac{x}{2{\left(Dt\right)}^{1/2}}\right)$$

where C(x) stands for the concentration at a given coordinate *x* and time t; $C_{i}$ is the concentration for x<0 at t=0; $C_{0}$ is the concentration at t=0 and x>0; D is the diffusion coefficient; t is the aging time. 

*D* was calculated by the relation $D=1/m^{2}$, where m is the slope of the curve obtained by plotting:

(5) $$erfc^{-1}\left(2\frac{C\left(x\right)-C_{0}}{C_{i}-C_{0}}\right)\;vs.\;\left(\frac{x}{2{\left(t\right)}^{1/2}}\right)$$

For both Cr and Fe, the diffusion coefficient value resulted as 2.5 × 10^−15^ cm^2^ s^−1^. The diffusion length over time was calculated using such a result for the whole experiment duration (Figure 3b). This parameter increased following a power law in agreement with the low-frequency process (P-II) of the impedance. The formation of an interaction layer is expected to increase the local conductivity of the glass by enrichment in transition elements [48,49] and formation of conducting phases [50]. Therefore, the observed increase in P-II conductivity could be ascribed to the Fe and Cr enrichment and the ${\mathrm{Cr}}_{2}O_{3}$ formation, as discussed before. 

From the morphological point of view, the substrate/sealant interface evolved over time, showing an increase in steel roughness (Figure 5) as a result of the high-temperature oxidation [23,51] promoted by the testing conditions. The roughness ratio of the substrate, calculated on samples as sintered, aged for 100 h and 250 h, showed an increase over time. This feature evolved following a power law trend (Figure 3d) as the conductivity of the low-frequency process (P-II, Figure 3a). The higher roughness increased the extension of the substrate/sealant interface, consequently reducing the interfacial electrical resistance.

The P-II trend showed a deviation from the power law between 40 and 160 h, highlighted by empty markers in Figure 3a (range excluded for fitting). The reason for this deviation is possibly due to the effect of the two identified interfacial processes having the same trend but different rate. Further investigations are needed to clarify this point, attributing properly the effect of each phenomenon.

**Figure 6:**
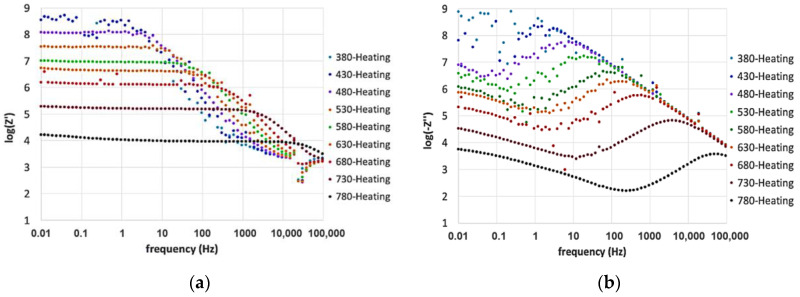
Frequency dependence of (**a**) the real part, Z’, and (**b**) the imaginary part, −Z’’, measured in the temperature range of 380–780 °C, during heating.

**Figure 7:**
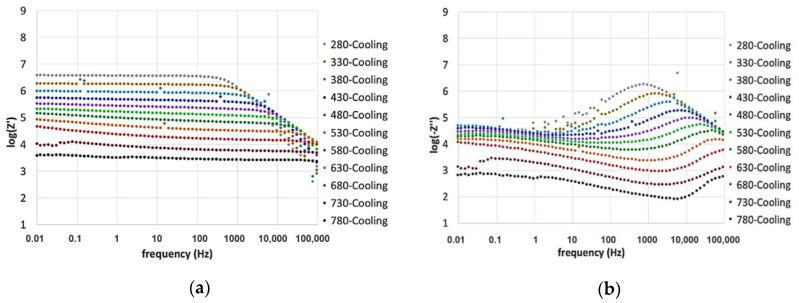
Frequency dependence of (**a**) the real part, Z’, and (**b**) the imaginary part, −Z’’, measured in the temperature range of 280–780 °C, during cooling.

Figure 6 and Figure 7 report the dependence of both real and imaginary parts of EIS on frequency and temperature. Measures were carried out while heating the sample before aging at 780 °C, and cooling after 250 h. 

Noise affected low-temperature measurements, especially the imaginary part of the impedance. It improved when the resistance decreased as an effect of temperature or aging at 780 °C.

The real part of the impedance was characterized by a plateau at low frequencies, which can be associated with the DC resistance of the glass. This value depends on the charge carriers’ diffusion via an activated hopping process [24,40,52,53]. 

At high frequencies, the impedance decreased linearly in a range attributed to the AC resistance as commonly observed for glasses. Such behavior is attributable to the localized motion of ions promoting the dipolar relaxation observable in the frequency domain [33,40,52,53]. 

After increasing the temperature, the plateau in the real part Z’ decreased, and the onset of the frequency-dependent region shifted towards higher frequencies, indicating a semiconductor behavior with thermally activated conduction mechanisms [25,48,54]. The low-frequency dispersion observed at low frequencies, due to the electrode polarization phenomena, appeared above 530 °C and increased with the temperature. 

At the highest temperatures, DC conduction becomes the dominant mechanism as indicated by the inclusion of the high-frequency contribution in the Z’ plateau [26]. In fact, the low-frequency conductivity resulted to be more temperature-dependent than the high-frequency one. 

The imaginary part of the impedance (Figure 6b and Figure 7b) showed a local maximum, centered at the high-frequency dispersion region of the Z’ contribution, related to relaxation processes. The frequency region below the local maximum represents data related to the long-distance mobility of the charge carriers. At higher frequencies, ions can only move within their potential wells and are blocked into the glass structure due to rapid inversions of the electric field direction [26,33,40].

The local maximum height in the imaginary part is decreasing and shifting toward high frequencies when increasing the temperature. The agreement with what was observed for Z’ confirms the presence of temperature-dependent relaxation processes and highlights their close relation with the DC conductivity [26].

DC conductivity of the glass-ceramic at 780 °C was determined with Equation (3) using the plateau value in the real part of the impedance. It increased from 1 × 10^−6^ to 5 × 10^−6^ S∙cm^−1^ as a consequence of aging for 250 h. The increase in the electrical conductivity was coherent with the evolution of the impedance contribution and the microstructural changes observed by post-experiment characterization. Despite the measured increase, the resulting value remained more than one order of magnitude below the maximum limit (1 × 10^−4^ S∙cm^−1^) [27]. Considering the stabilizing trend of the microstructural processes influencing the conductivity, its value is expected to not reach the recommended limit for the entire operating time of an SOFC stack.

**Figure 8:**
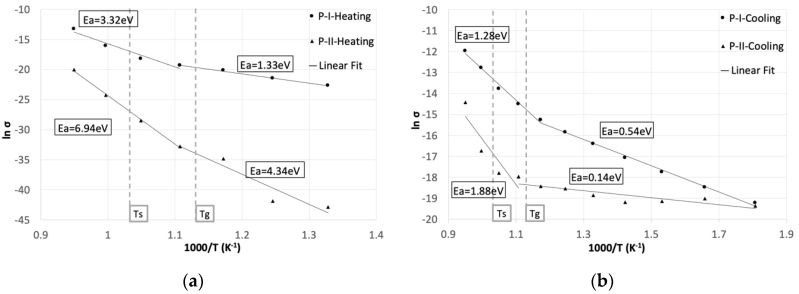
Temperature dependence of the specific conductivities for the high (P-I)- and low (P-II)-frequency processes (**a**) during heating and (**b**) cooling after aging for 250 h at 780 °C. Labels report the activation energies of the corresponding segments. Dashed lines indicate the softening temperature (Ts) and the glass transition temperature (Tg).

The specific conductivities, calculated using Equation (3) from the impedance contributions at different temperatures, are reported in the Arrhenius plot in Figure 8. According to the observations in Figure 6 and Figure 7, the high-frequency process (P-I in Figure 8) showed the higher conductivity values for all the temperatures, while the low-frequency contribution (P-II in Figure 8) resulted as the most temperature-activated, considering its slope during heating. The observed temperature dependence reflected the thermally activated nature of ionic hopping processes [40] and can be described by the Arrhenius equation:

(6) $$\sigma =\sigma_{0}e^{\frac{-E_{a}}{kT}}$$

where *σ*_0_ is the pre-exponential factor, $E_{a}$ is the activation energy of the process and k and T stand for the Boltzmann constant and the temperature in kelvin, respectively. Values of *E_a_* were calculated from the slopes of the linear fits of the curves in Figure 8. 

Both processes showed a rise in the activation energy above the Tg, where the influence of temperature on the conductivity increased. This effect can be attributed to the gradual decrease in viscosity of the glass above the Tg, facilitating the ion movements induced by the application of the external electric field [26,27,39,43,52,53]. Additionally, the higher influence of temperature on the low-frequency process can be attributed to the twofold effect of the lower viscosity of the glass: enhancing the ionic conductivity and increasing the mechanical contact between the glass and the steel. 

The activation energies measured after aging were lower for both processes due to the microstructural changes that occurred at the interfaces and in the bulk. 

The decrease in the activation energy measured at low frequencies can be attributed to the improved conductivity at the interface, resulting from the enhanced contact and diffusion processes.

The high-frequency process, related to the bulk crystallinity, exhibited a lower activation energy after aging, indicating an easier ionic transport in the crystallized glass. This effect is in agreement with the hypothesis of the increased concentration of conductive species in the residual glassy matrix [33,55]. A second theory supports that the conductivity is higher at the boundaries between the residual glass phase and crystals [25], so it should increase with the extension of the crystal/glass interface. However, the observed changes in crystallinity were due to the growth of already formed crystals, therefore decreasing the total phase boundary and confirming the first hypothesis.

## 4. Conclusions

In this work, the behavior of a glass-ceramic composition in contact with FSS substrates was investigated by means of EIS. Such a technique turned out to be suitable to study the effects of microstructural changes on the electrical properties of sealing materials at SOFC stack working temperatures.

Two thermally activated impedance contributions were measured and compared with post-experiment evidence. The high-frequency process resulted to be less temperature-dependent and related to AC conduction phenomena, due to the short-distance movement of ions. The low-frequency contribution resulted to be strongly thermally dependent and was attributed to the DC conductivity, related to the long-range migration of mobile ions.

Both processes were affected by microstructural changes occurring as an effect of aging at SOFC operating temperature. The high-frequency process evolved over time showing a decrease in its associated electrical resistance. The aging conditions promoted a progressive crystallization of the glass-ceramic causing the increase in concentration of the conductive species in the residual glassy matrix, enhancing the overall glass conductivity. 

At the same time, the low-frequency process was influenced by the evolution of the glass/steel interfaces. The related electrical resistance decreased over time as a result of the extension of the contact interface between the two materials and the formation of an interaction layer rich in chromium and iron, locally increasing the electrical conductivity of the glass.

The DC conductivity of the glass-ceramic increased coherently with the impedance contribution and the microstructural changes observed during the experiment time. The resulting value of the conductivity remained far below the maximum recommended limit that is supposed to not be exceeded for the entire operating life of an SOFC stack, considering the stabilizing trend of the related processes. 

The electrical characterization of glass-ceramics at SOFC operating conditions, in terms of interfaces and temperature, is crucial to adequately choose the sealing material. The evolution of conductivity processes over time, related to microstructural changes, allows to study the sealant degradation processes providing useful information for the material development. Additionally, such a method could be further adapted to monitor, in a non-destructive way, the state of the sealant in stacks.

## Figures and Tables

**Table 1:** Nominal composition of AISI 441.

**Element**	Fe	C	Si	Mn	Cr	Ti + Nb
**Concentration (wt.%)**	Balance	0.015	0.60	0.30	17.80	0.65
